# Prevalence and genetic diversity of multidrug-resistant *Salmonella* Typhimurium monophasic variant in a swine farm from China

**DOI:** 10.3389/fmicb.2023.1200088

**Published:** 2023-06-15

**Authors:** Lin Zhou, Tie-Jun Zhang, Weicheng Zhang, Chengjiang Xie, Ye Yang, Xuan Chen, Qin Wang, Hong-Ning Wang, Chang-Wei Lei

**Affiliations:** Key Laboratory of Bio-Resource and Eco-Environment of Ministry of Education, Animal Disease Prevention and Food Safety Key Laboratory of Sichuan Province, College of Life Sciences, Sichuan University, Chengdu, China

**Keywords:** *Salmonella*, whole-genome sequencing, multidrug resistance, IncHI2 plasmids, *mcr-1*, 4,[5],12:i:-

## Abstract

*Salmonella* 4,[5],12:i:-, a monophasic variant of *S*. Typhimurium, has become a global serovar causing animal and human infections since its first emergence in the late 1980's. Several previous studies showed the increasing prevalence of *S*. 4,[5],12:i:- in China, most of which were from swine with multidrug resistance (MDR) profiles. However, the molecular characteristic and evolution of *S*. 4,[5],12:i:- in the same swine farm are still unknown. In this study, a total of 54 *S. enterica* strains were isolated from different fattening pigs aged 1, 3, and 6 months, most of which belonged to *S*. 4,[5],12:i:-. Whole-genome sequencing revealed that all 45 *S*. 4,[5],12:i:- strains belonged to ST34 and were further divided into two different ribosomal STs and nine different core-genome STs. Phylogenetic analysis of 286 *S*. 4,[5],12:i:- strains in China, including 241 from the EnteroBase *Salmonella* database, revealed the genetic diversity of *S*. 4,[5],12:i:- and indicated that *S*. 4,[5],12:i:- in this swine farm might have multiple origins. Three different IncHI2 plasmids carrying various resistance genes were characterized by nanopore sequencing and could be conjugated to *Escherichia coli*. The colistin resistance gene *mcr-1* and ESBLs gene *bla*_CTX − M−14_ were co-located on the chromosome of one strain. The dynamic changes in antimicrobial resistance regions and transferability of IncHI2 plasmids, as well as the chromosomal location of resistance genes, facilitated the diversity of the antimicrobial resistance characteristics in *S*. 4,[5],12:i:-. Since the swine farm is regarded as the important reservoir of MDR *S*. 4,[5],12:i:-, the prevalence and evolution of *S*. 4,[5],12:i:- from swine farms to pig products and humans should be continually monitored.

## Introduction

*Salmonella* is regarded as a type of gram-negative bacterium with one of the most important zoonotic and foodborne pathogens (Igbinosa et al., 2021). Infectious diseases caused by *Salmonella* have recently been at the center of attention for food practitioners worldwide (Tian et al., 2021). According to the WHO, 9% of diarrheal illnesses are caused by *Salmonella* globally each year (Besser, 2018). Data from China report that over 70% of bacterial food poisoning are relevant to *Salmonella*, which means that it brings a huge challenge to food safety (Shen et al., 2022). *Salmonella enterica* serovar Typhimurium (*S*. Typhimurium) is one of the most frequent serovars related to foodborne diseases (Sun et al., 2020). *S*. Typhimurium is a non-typhoidal *Salmonella*, and this pathogen may cause enteritis. In the United States and Europe, it can spread in the cold chain and is responsible for many foodborne disease outbreaks caused by pigs and pig products (Bonardi, 2017).

The past two decades have witnessed a rapid worldwide emergence of a new *Salmonella* serovar, a monophasic variant of *S*. Typhimurium, whose antigenic formula is 1,4,[5],12:i:- (Ingle et al., 2021). This type of strain lacks an expression of phase 2 flagella, which may be due to different mutations (including point mutations) and partial or complete deletions in *fljB* and adjacent genes (Zamperini et al., 2007). Some strains have IncHI2 plasmids carrying mobilized colistin resistance gene *mcr* and ESBL genes, as well as heavy metal resistance (Diaconu et al., 2021). A high multidrug resistance (MDR) rate was present in *S*. 1,4,[5],12:i:-, with the profile of resistance to ampicillin, streptomycin, sulfonamides, and tetracycline, i.e., ASSuT pattern, largely dominating in China (Qin et al., 2022).

It has been confirmed that pig meat and pork meat are the main sources of *S*. 1,4,[5],12:i:- (Hauser et al., 2010; Ferrari et al., 2019), and *S*. 1,4,[5],12:i:- is strongly associated with the swine food chain, especially in Europe and the United States, suggesting a potential link between human infections with this serovar and pork and pork products in these areas (Sun et al., 2020). Most *S*. 1,4,[5],12:i:- strains (48%) in Germany were isolated from pork between 2006 and 2008 (Hauser et al., 2010). *S*. 4,[5],12:i:- has been reported to be the second most frequently encountered serovar in patients in Henan province, China (Sun et al., 2020). Several previous studies showed the increasing prevalence of *S*. 4,[5],12:i:- in China (Mu et al., 2022), most of which were from swine with high resistance rates (Xie et al., 2020; Shen et al., 2022). However, the molecular characteristic and evolution of *S*. 4,[5],12:i:- in the same swine farm are still unknown. In this study, we determined the genetic relationship and antimicrobial resistance of *Salmonella* and revealed the persistence and genetic evolution of MDR *S*. 4,[5],12:i:- in a swine farm in China.

## Materials and methods

### Sample collection and *Salmonella* isolation

The large-scale swine farm (~10,000 fattening pigs) is located in Sichuan province, China. The piglets were fed at 21–25°C, and the fattening pigs were fed at 18–23°C, which were vaccinated and in healthy condition. The piglets were fed with florfenicol or doxycycline to prevent diarrhea. A total of 450 fresh fecal swabs from different fattening pigs aged between ~1, 3, and 6 months (150 fattening pigs at each stage) were randomly collected between October and November 2021. *Salmonella* isolation was carried out as previously described (Yang et al., 2014). All culture mediums were purchased from Qingdao Hope Bio Technology, China. The swab samples were inoculated into 3 ml of buffer peptone water (BPW). Then, the 0.5 ml BPW-incubated culture was transferred to 10 ml of tetrathionate (TT) broth, and another 0.1 ml was transferred to 10 ml of Rappaport-Vassiliadis (RV) enrichment broth incubated at 42°C for 24 h. One loopful of each TT and RV broth culture was streaked onto xylose lysine tergitol 4 (XLT4) and brilliant green sulfa (BGS) agar and incubated at 37°C for 24 h. In the case of growth, one typical *Salmonella* colony was randomly selected from each plate and confirmed using the BD Phoenix-100 system (Sparks, MD, United States). Only one isolate from each sample was selected for further study.

### Antimicrobial susceptibility testing

Susceptibilities to 16 antimicrobial agents, including ampicillin (AMP), amoxicillin-clavulanate (AMC), ceftriaxone (CRO), ceftazidime (CAZ), meropenem (MEM), streptomycin (STR), gentamicin (GEN), amikacin (AMK), ciprofloxacin (CIP), norfloxacin (NOR), chloramphenicol (CHL), florfenicol (FFC), sulfamethoxazole (SUL), trimethoprim/sulfamethoxazole (SXT), tetracycline (TET), and polymyxin B (PMB), were determined using the standard Kirby–Bauer disk diffusion method according to Clinical and Laboratory Standards Institute guidelines (Clinical Laboratory Standards Institute, 2020). *Escherichia coli* ATCC25922 was used as a control strain.

### Whole-genome sequencing and bioinformatics analyses

Genomic DNAs of *Salmonella* isolates were extracted using a bacterial genomic DNA extraction kit (Tiangen, China). Whole-genome sequencing (WGS) was performed using the Illumina HiSeq 4000 platform (150 bp paired-end reads with ~200-fold average coverage). The raw reads were uploaded to the EnteroBase *Salmonella* database (http://enterobase.warwick.ac.uk/species/index/senterica) for genome assembly, serotyping, multilocus sequence typing (MLST; Maiden et al., 1998), ribosomal MLST (rMLST; Jolley et al., 2012), and core-genome MLST (cgMLST; Alikhan et al., 2018). The prokaryotic genome annotation software, RAST (Aziz et al., 2008), was used to identify the basic information of the draft genomes, such as the size of the genomes, GC content (%), and the number of contigs (above 200 bp). Antimicrobial resistance genes and plasmids were identified by CGE ResFinder 4.1 (Bortolaia et al., 2020) and PlasmidFinder 2.0 (Carattoli et al., 2014), respectively.

### Phylogenetic analysis of *S*. 4,[5],12:i:- strains in China

To interpret the phylogenetic relationship of the *S*. 4,[5],12:i:- strains in China, the phylogenetic tree based on SNPs was constructed using the EnteroBase *Salmonella* database combining 45 strains in this study and 241 strains in the database (accessed 3 January 2023). The phylogenetic tree was modified in Itol (Letunic and Bork, 2021). The presence of *Salmonella* genomic island 4 (SGI4) was identified by the BLAST program (Camacho et al., 2009), using the SGI4 sequence in *S*. Typhimurium strain SLK-532 (GenBank accession number MN730129) as the reference sequence (Mourao et al., 2020).

### Plasmid assembly, BLAST analysis, and conjugation assays

Three *S*. 4,[5],12:i:- strains, namely 3M-23, 6M-54, and 6M-71, harboring IncHI2 plasmids and various resistance genes were further sequenced by Nanopore MinION using Rapid Sequencing Kit. The complete genome sequences were assembled by Unicycler v0.5.0 using nanopore sequencing data combined with Illumina sequencing data. The complete nucleotide sequences of IncHI2 plasmids and the *mcr-1* region were analyzed using the BLAST program (Camacho et al., 2009). BLAST Ring Image Generator (BRIG; Alikhan et al., 2011) and Easyfig v2.2.2 (Sullivan et al., 2011) were used for comparative analyses of the genetic environments. Conjugation experiments were performed by using 3M-23, 6M-54, and 6M-71 as the donor strains and rifampin-resistant *E. coli* EC600 as the recipient strain with selection on nutrient agar plates containing 300 mg/l rifampin and 8 mg/l florfenicol. The positive transconjugants were further determined by 16S rRNA gene sequencing and detection of antimicrobial resistance profiles.

### Nucleotide sequence accession numbers

The genomes of the 54 *S*. *enterica* isolates reported in this study have been deposited in National Center for Biotechnology Information and registered BioProject number PRJNA912362. The complete nucleotide sequences of three IncHI2 plasmids and the *mcr-1* region in 6M-54 have been deposited in GenBank and assigned accession numbers OP970992-OP970994 and OP978507.

## Results

### Prevalence of *S. enterica* in the swine farm

A total of 54 *S. enterica* strains were isolated and identified from 450 samples, with an isolation rate of 12%. Most of the strains (83.3%, 45/54) were recovered from the 6-month fattening pigs, and only two strains came from the 3-month fattening pigs. Serotyping based on the genome sequences indicated that those strains belonged to three serovars, 4,[5],12:i:- (the monophasic variant of Typhimurium; *n* = 45), Enteritidis (*n* = 7), and Derby (*n* = 2; Table 1). All 7 *S*. Enteritidis strains came from the 1-month pigs, and 43 out of 45 *S*. 4,[5],12:i:- strains came from the 6-month fattening pigs. Detailed information on the 54 *S. enterica* strains reported in this study is presented in Supplementary Table 1.

**Table 1:** Characterization of *Salmonella enterica* strains in the swine farm.

**Serovar**	**ST**	**rST**	**Resistance genes**	**Resistant plasmid(s)**	**Number of strains**
I 1, 4,[5],12:i:- (*n* = 45)	34	1,369	*aph(6)-Id, aph(3′')-Ib, sul2*, *bla*_TEM − 1B_, and *tet*(B)	IncQ1	39
			*aac(3)-IV, aph(6)-Id, aph(3′')-Ib, aph(3′)-Ia, aph(4)-Ia, aadA1*, *bla*_OXA − 10_, *bla*_TEM − 1B_, *tet*(A), *tet*(B), *arr-2, qnrS1, sul2, dfrA14, floR, cmlA1*, and *lnu(F)*	p6M-71 and IncQ1	2
			*aac(3)-IV, aph(6)-Id, aph(3′')-Ib, aph(3′)-Ia, aph(4)-Ia, aadA1*, *bla*_OXA − 10_, *bla*_TEM − 1B_, *tet*(A), *arr-2, qnrS1, sul2, dfrA14, floR, cmlA1*, and *lnu(F)*	p6M-71 and IncQ1	1
			*aac(3)-IV, aph(6)-Id, aph(3′')-Ib, aph(3′)-Ia, aph(4)-Ia, aac(6′)-Ib-cr, aadA1, aadA2b, mcr-1.1*, *bla*_OXA − 1_, *bla*_CTX − M−14_, *tet*(B), *qnrS2, dfrA12, sul1, sul2, sul3, arr-3, floR, catB3*, and *cmlA1*	p6M-54 and IncQ1	1
		60,594	*aac(3)-IV, aph(6)-Id, aph(3′')-Ib, aph(3′)-Ia, aph(4)-Ia, aadA1, dfrA14, sul2, qnrS1, tet*(A), *bla*_TEM − 1B_, *bla*_CTX − M−65_, *bla*_OXA − 10_, *arr-2, floR, cmlA1*, and *lnu(F)*	p3M-23 and IncQ1	2
Enteritidis (*n* = 7)	11	1,425	-	-	7
Derby (*n* = 2)	40	3,735	*fosA7* and *qnrS1*	-	2

### Molecular typing of *S. enterica* and phylogenetic analysis of *S*. 4,[5],12:i:-

In total, 45 *S*. 4,[5],12:i:- strains belonged to ST34 and were further divided into two different rSTs, namely rST1369 and rST60594. It was worth noting that 43 strains from the 6-month fattening pigs belonged to rST1369, and the other two strains from the 3-month fattening pigs belonged to rST60594. The 45 *S*. 4,[5],12:i:- strains consisted of nine different cgSTs, highlighting the genetic diversity and presence of different lineages of *S*. 4,[5],12:i:- in the same farm. The cgST303338 (*n* = 35) was the predominant lineage. All seven *S*. Enteritidis strains belonged to ST11 and shared the same rST1425 and cgST303335, indicating that those strains were clonally related. The two *S*. Derby strains belonged to ST40 and rST3735 and were divided into two different cgSTs, namely 303905 and 303918 (Supplementary Table 1).

**Figure 1:**
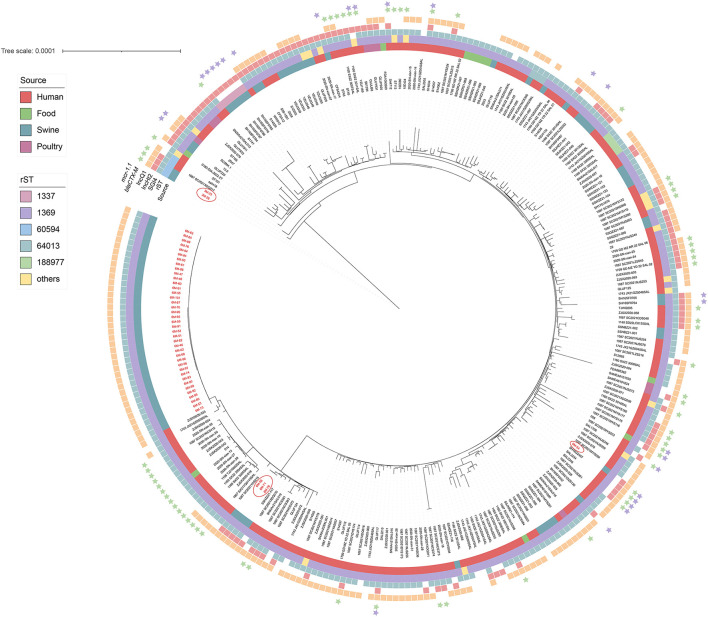
Phylogenetic tree based on the SNPs for the 286 *S*. 4,[5],12:i:- strains in China. The different sources and rSTs, as well as the presence of plasmids, *Salmonella* genomic island 4 (SGI4), *mcr-1*, and *bla*_CTX − M_ genes are indicated in different colors. The names of the strains reported in this study are in red.

To investigate the phylogenetic relationship of these 45 *S*. 4,[5],12:i:- isolates, a maximum likelihood core-genome phylogenetic tree was constructed using 286 *S*. 4,[5],12:i:- strains (241 from EnteroBase *Salmonella* database and 45 from this study), with different sources (human, *n* = 179; swine, *n* = 87; food, *n* = 11; and poultry, *n* = 9) in China based on 4,243 SNPs (between 0 and 646 pairwise SNPs; median = 59; Figure 1). The pairwise SNP distance of 45 *S*. 4,[5],12:i:- strains from this study was between 0 and 560; however, the SNP distance of 39 strains was between 0 and 5. Furthermore, these 39 strains were divided into six cgSTs (cgST303338, *n* = 33, token the majority). The minimum SNPs distance between human isolates and pig isolates was 12 (6M-71/6M-78 from pigs and SSHZZ21-117 from humans). All 286 *S*. 4,[5],12:i:- strains belonged to ST34 and were divided into 22 rSTs, in which rST1369 (87.4%, 250/286) was the predominant rST (Supplementary Table 2). In total, 45 strains in this study were located in four different positions of the phylogenetic tree, especially strains 3M-23 and 3M-26 that belonged to rST60594 were in the root position (Figure 1). It was found that 90.6% (259/286) strains harbored SGI4, an integrative and conjugative element that carried heavy metal tolerance genes showing resistance to silver, arsenic, and copper (Branchu et al., 2019). The IncQ1 replicon gene was detected in 76.2% (218/286) strains; on the other hand, IncHI2 and IncHI2A replicon genes were detected in 37.4% (107/286) strains. The most commonly acquired AMR gene detected among these strains was *aac(6*′*)-Iaa* (286/286, encoding resistance to tobramycin and amikacin), followed by *sul2* (246/286, sulfamethoxazole), *tet*(B) (244/286, tetracycline), *aph(6)-Id* (235/286, streptomycin), *aph(3*′'*)-Ib* (222/286, streptomycin), and *bla*_TEM − 1B_ (178/286, beta-lactam). Three *bla*_CTX − M_ subtypes were identified among these strains, including *bla*_CTX − M−14_ (13/286), *bla*_CTX − M−55_ (26/286), and *bla*_CTX − M−65_ (28/286). *fosA3* encoding for fosfomycin resistance was detected in seven strains. In addition, mobile colistin resistance gene *mcr-1* was detected in 26 strains, 14 (53.8%) of them were of pig origin. It was also found that all of the *mcr-1* positive strains detected IncHI2 and IncHI2A replicon genes.

### Antimicrobial resistance profiles of *S. enterica*

Antimicrobial susceptibility testing showed that the 45 *S*. 4,[5],12:i:- strains displayed four different resistance profiles, and all exhibited ASSuT resistance patterns (ampicillin, streptomycin, sulfonamides, and tetracycline; Hopkins et al., 2010). In addition, six strains were resistant to SXT, GEN, CHL, and FFC. Three strains showed resistance to CRO, one of which was also resistant to PMB. The seven *S*. Enteritidis and two *S*. Derby strains were susceptible to all 16 antimicrobial agents tested. Five different antimicrobial resistance genotypes were identified in *S*. 4,[5],12:i:- strains (Table 1). Comparative genomic analysis showed that the IncQ1 plasmid carrying *bla*_TEM − 1B_, *aph(3*′'*)-Ib* (*strA*), *aph(6)-Id* (*strB*), *sul2*, and *tet*(B) was inserted in the chromosomal *fljBA* operon. However, three strains lost the *tet*(B) gene. Two strains (3M-23 and 3M-26) harbored ESBLs gene *bla*_CTX − M−65_, and one strain (6M-54) harbored ESBLs gene *bla*_CTX − M−14_, fluoroquinolone resistance gene *aac(6*′*)-Ib-cr*, and colistin resistance gene *mcr-1* (Supplementary Table 1).

### Antimicrobial-resistant IncHI2 plasmids in *S*. 4,[5],12:i:-

**Figure 2:**
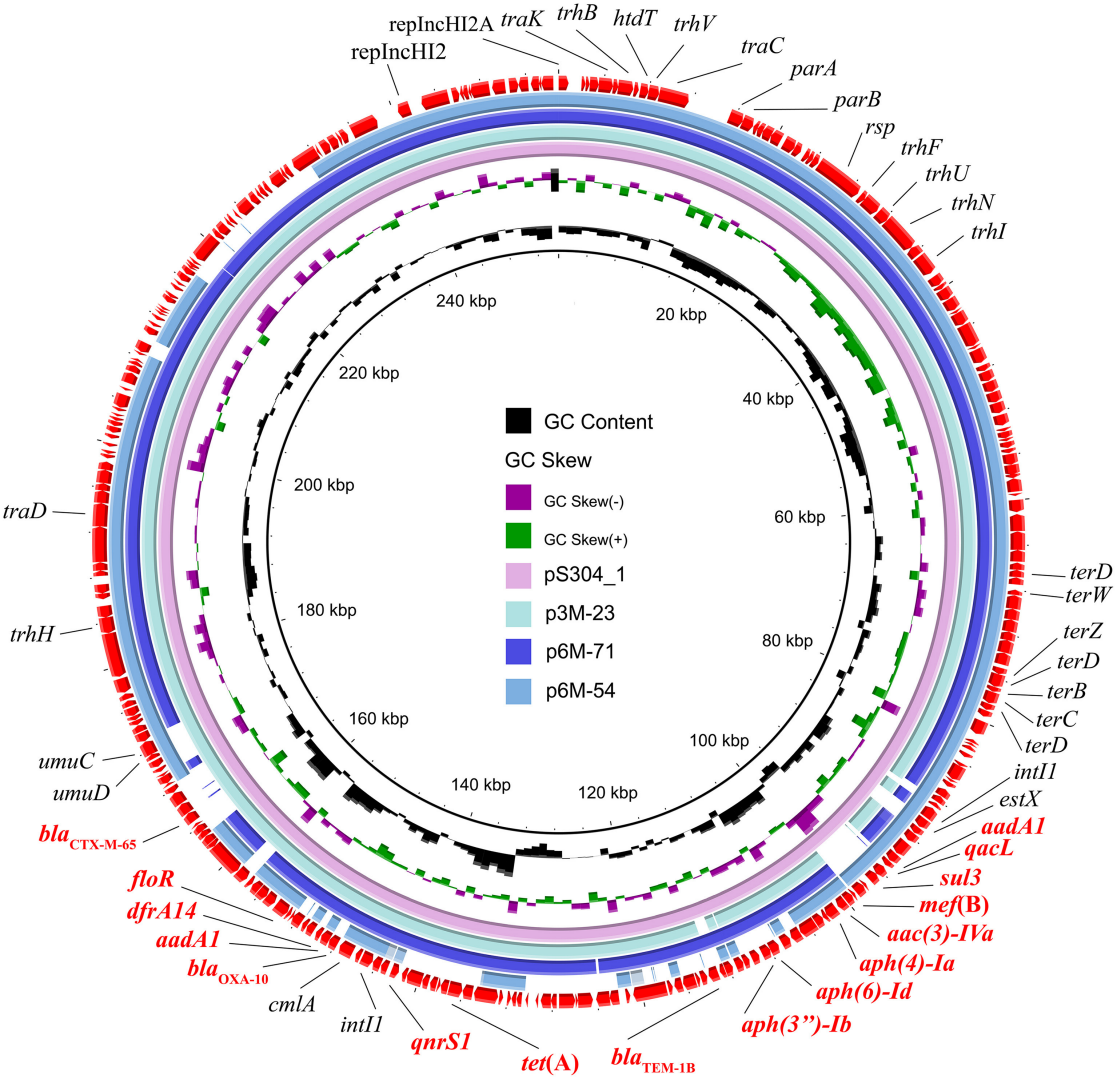
Circular representation of the three IncHI2 plasmids. The comparison among p3M-23 (OP970992), p6M-71 (OP970994), and p6M-54 (OP970993) with pS304_1 (CP061127) was generated using BRIG v0.95.

**Figure 3:**
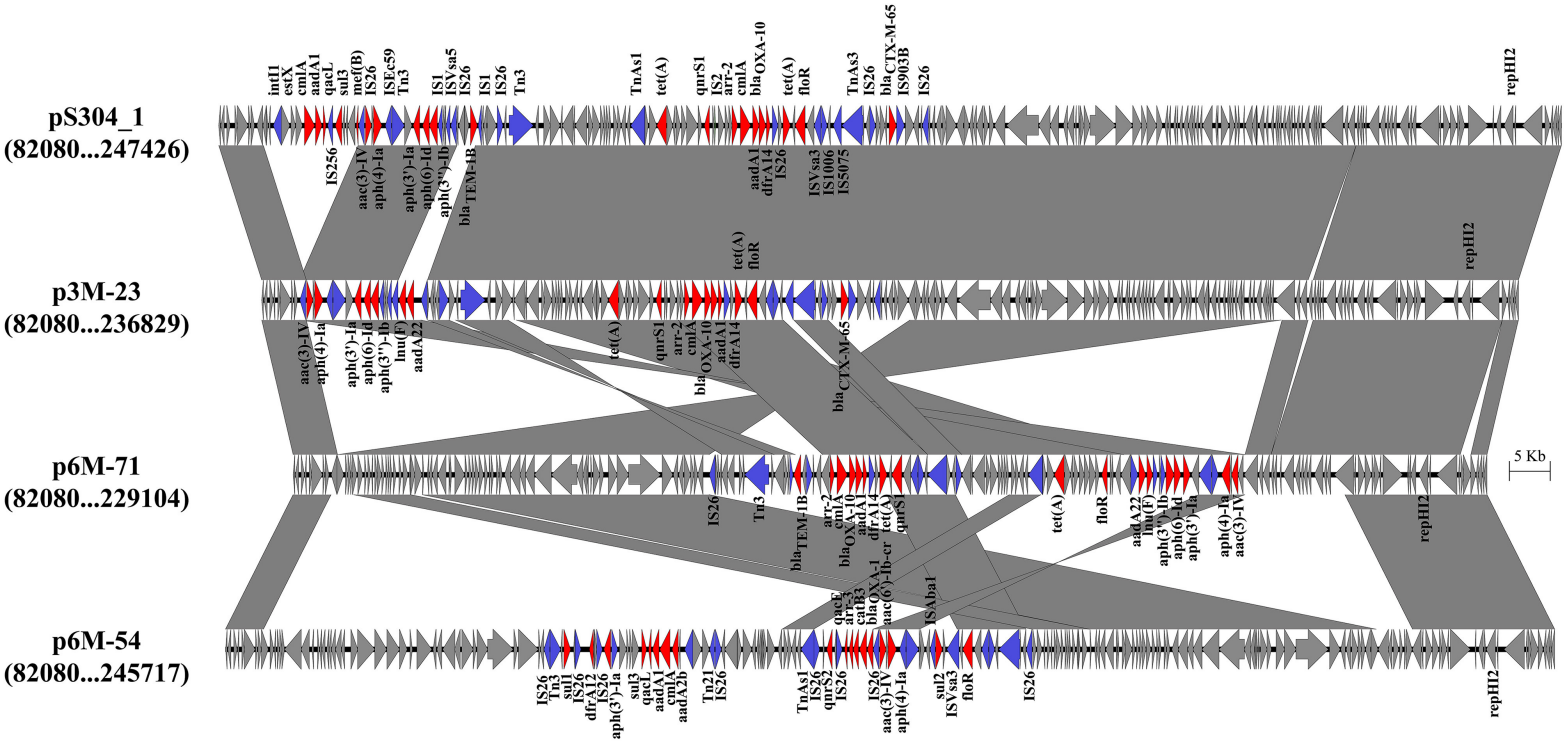
Genetic structures of the antimicrobial resistance regions in the IncHI2 plasmids. Genes and ORFs are shown as arrows, and their orientations of transcription are indicated by the arrowheads. Shared regions with above 99% identity are indicated by shading. Antimicrobial resistance genes and transposase or integrase genes are in red and blue, respectively.

Three different IncHI2 plasmids, namely p3M-23, p6M-71, and p6M-54, were characterized in this study (Figure 2). The sizes of the three plasmids varied from 236,551 to 253,164 bp. BLAST analysis showed that they shared ~96-kb conservative backbone region carrying functional genes including replication gene, *tra/trh* genes associated with the conjugative transfer, and tellurium resistance gene cluster (Figure 2). p3M-23 shared 99.99% nucleotide identity with 99% cover to pS304_1 (CP061127), an IncHI2 plasmid in *S*. Typhimurium strain recovered from the stool of a patient in China. A comparative analysis of the antimicrobial resistance regions in the three IncHI2 plasmids is shown in Figure 3. The mosaic antimicrobial resistance regions consisted of various resistance genes, insertion sequences, and transposons. Compared with pS304_1, p3M-23 lost class I integron region *intI1*-*estX*-*cmlA*-*aadA1*-*qacL*-*sul3*, and the IS*26*-*lun*(F)-*aadA22*-IS*1*-IS*26* region was instead of IS*26*-*bla*_TEM − 1B_-IS*1*-IS*26* region (Figure 3). p3M-23 and p6M-71 shared the same 16 resistance genes, but p3M-23 supplemented *bla*_CTX − M−65_ gene flanked by IS*26* and IS*903B*. The antimicrobial resistance regions in p6M-54 showed a high difference from those in p3M-23 and p6M-71. Conjugation experiments showed that the three IncHI2 plasmids reported in this study could be easily conjugated to *E. coli*.

**Figure 4:**
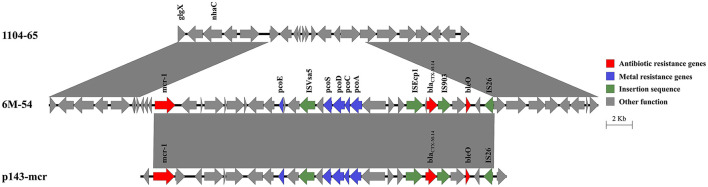
Genetic structure of the *mcr-1* and *bla*_CTX − M−14_ regions in the chromosome of strain 6M-54. Structures are drawn to scale from GenBank accession numbers CP110201 (strain 1104-65), OP978507 (strain 6M-54), and CP091564 (p143-mcr).

The *mcr-1* and *bla*_CTX − M−14_, as well as a copper resistance gene cluster, were co-located on the chromosome of 6M-54 (Figure 4). The *bla*_CTX − M−14_ gene was flanked by IS*Ecp1* and IS*903*. The integration of this 27.3-kb region into the chromosome resulted in a deletion of the 7.4-kb chromosomal segment. BLAST analysis showed that it shared the 26.2-kb region with IncHI2 plasmid p143-mcr (CP091564) with 100% nucleotide identity, speculating that this region harboring *mcr-1* and *bla*_CTX − M−14_ might come from the IncHI2 plasmid.

## Discussion

In this study, we determined the prevalence and genetic diversity of MDR *S*. 4,[5],12:i:- in a swine farm in China. *S*. 4,[5],12:i:- has rapidly emerged worldwide in the last two decades and has become an important serovar of *Salmonella* associated with human salmonellosis. Epidemiological data indicated that the incidence of *S*. 4,[5],12:i:- was related to the pig food chain (European Food Safety Authority European Centre for Disease Prevention Control, 2017), and *S*. 4,[5],12:i:- could be transmitted through the food chain between humans and animals. In this study, 54 *S. enterica* strains were isolated from 450 samples; of which, 45 belonged to *S*. 4,[5],12:i:-. A study by He et al. (2020) reported that 255 *S*. 4,[5],12:i:- isolates were collected from 10 provinces in China from 2010 to 2018, in which 222 (87.1%) were obtained from swine. Another study based on CRISPR typing revealed that the pig was considered as a main reservoir for *S*. 4,[5],12:i:- (Xie et al., 2020). These results indicate that *S*. 4,[5],12:i:- is prevalent in swine farms in China.

Our study confirms that WGS is an efficient molecular typing in the monitoring of *Salmonella* (Tang et al., 2019). Phylogenetic analysis based on the genome SNPs indicated that the 286 *S*. 4,[5],12:i:- strains in China showed high genetic diversity. In this study, we found that 39 out of the 45 *S*. 4,[5],12:i:- were clonally related (0–5 SNPs), which formed a predominant clone in this swine farm. In addition, we also found that there were strains belonging to multiple different lineages in this swine farm, especially two strains (6M-71 and 6M-78) were very close to human isolate SSHZZ21-117 (12 SNPs). The genetic diversity indicated that *S*. 4,[5],12:i:- in this swine farm might have multiple origins.

We found that 45 *S*. 4,[5],12:i:- isolated in this farm displayed MDR profiles and harbored various resistance genes. It has been reported that 91.8% (234/255) isolates in China were MDR (He et al., 2020), suggesting that multiple antimicrobial resistance is a common phenomenon in *S*. 4,[5],12:i:- isolates. The chromosomal inserted IncQ1 plasmid in *S*. 4,[5],12:i:- often carried five resistance genes, namely *bla*_TEM − 1B_, *aph(3*′'*)-Ib, aph(6)-Id, sul2*, and *tet*(B), mediating resistance to beta-lactams, aminoglycosides, sulfonamides, and tetracyclines. The extensive use of doxycycline belonging to tetracyclines in large-scale swine farms may promote the persistence of *S*. 4,[5],12:i:-. In addition, we found that two strains harbored ESBLs gene *bla*_CTX − M−65_, and one strain harbored ESBLs gene *bla*_CTX − M−14_ and colistin resistance gene *mcr-1*. The prevalence of clinically important antimicrobial resistance genes in *S*. 4,[5],12:i:-, such as ESBLs and colistin resistance genes (Diaconu et al., 2021), poses a serious threat to public health.

Mobile genetic elements, such as plasmids and insertion sequences, play a central role in facilitating the acquisition and spread of resistance genes (Partridge et al., 2018). It has been found that *S*. 4,[5],12:i:- strains were able to acquire the IncHI2 plasmids that harbored various resistance genes conferring resistance to multiple antimicrobial agents (Ingle et al., 2021; Mu et al., 2022). In this study, we characterized three different IncHI2 plasmids that carried 17–18 resistance genes. It was found that 6–8 copies of IS*26* were present in the antimicrobial resistance regions, highlighting that IS*26* played important roles in promoting the diversity of the IncHI2 plasmids (Harmer and Hall, 2016). It is very interesting to note that the *mcr-1* and *bla*_CTX − M−14_ were co-located on the chromosome of strain 6M-54, which might come from the IncHI2 plasmid (Li et al., 2017). The dynamic changes in antimicrobial resistance regions and transferability of the IncHI2 plasmids, as well as the chromosomal location of resistance genes, facilitated the diversity of the antimicrobial resistance phenotypes in *S*. 4,[5],12:i:-.

Our study indicates serious contamination of *S*. 4,[5],12:i:- in this large-scale swine farm, especially in the 6-month-old pigs that were close to slaughter, which may pose a serious threat to food safety and public health. Further study will be conducted to clarify the key elements, such as personnel activities, feed transportation, and overall environmental sanitation, which influence the prevalence of *S*. 4,[5],12:i:- in the swine farm. Hazard analysis and critical control point programs as well as strict bio-security measures should be implemented on swine farms to control *S*. 4,[5],12:i:-.

## Conclusion

Our study highlighted the prevalence and genetic diversity of MDR *S*. Typhimurium monophasic variant (*S*. 4,[5],12:i:-) in a swine farm in China by whole-genome sequencing. Phylogenetic analysis indicated that *S*. 4,[5],12:i:- in the swine farm reported in this study might have multiple origins. *S*. 4,[5],12:i:- displayed MDR profiles and harbored various resistance genes including clinically important antimicrobial resistance genes, such as ESBLs genes and colistin resistance gene *mcr-1*.The dynamic changes in antimicrobial resistance regions and transferability of the IncHI2 plasmids, as well as the chromosomal location of resistance genes, facilitated the diversity of the antimicrobial resistance characteristics in *S*. 4,[5],12:i:-. Since the swine farm is regarded as the important reservoir of MDR *S*. 4,[5],12:i:-, the prevalence and evolution of *S*. 4,[5],12:i:- from swine farms to pig products and humans should be continually monitored in terms of food safety and human health.

## Data availability statement

The datasets presented in this study can be found in online repositories. The names of the repository/repositories and accession number(s) can be found in the article/Supplementary material.

## Author contributions

LZ, T-JZ, H-NW, and C-WL: conception and design. LZ, WZ, CX, YY, and XC: methodology. T-JZ and QW: collection and assembly of data. LZ, T-JZ, and C-WL: data analysis and interpretation. LZ: writing—original draft. H-NW and C-WL: writing—reviewing and editing. All authors contributed to the article and approved the submitted version.

## Supplementary material

Supplementary Table 1 Detailed information on the 54 *S. enterica* strains reported in this study.

Supplementary Table 2 *S*. 4,[5],12:i:- in EnteroBase *Salmonella* database (accessed 3 January 2023).
